# Supplementary material for: NOXA Is Important for Verticillium dahliae’s Penetration Ability and Virulence
Source: J Fungi (Basel). 2021 Sep 28;7(10):814. doi: 10.3390/jof7100814 (PMC8541199; doi:10.3390/jof7100814)
Supplement: Supplementary file 1 [file jof-07-00814-s001.zip › jof-1361809-supplementary/Supplementary Table S1.pdf]

Supplementary Table S1. Primers used in confirmation of complemented strains

Primer's name	Primer sequence	Tm (°C)
NOXA-F-1-A	GTACGTGGTGAGGAAGCAG	58.5
NOXA-R-1-A	TTCAGTGTGTTGAGTGGCG	59.4
NOXA-F-2-A	GGTCTCGTGCTTAGTGTGG	57.6
NOXA-R-2-A	GAGATAACCGATGCAGTGC	59.6
NOXA-F-3-A	CCGCAGAGAGACTAAGATTAC	60.3
NOXA-R-3-A	CCGAGTTGAGGACGATGTT	58.3
