# Supplementary figures and images for: NOXA Is Important for Verticillium dahliae’s Penetration Ability and Virulence
Source: J Fungi (Basel). 2021 Sep 28;7(10):814. doi: 10.3390/jof7100814 (PMC8541199; doi:10.3390/jof7100814)

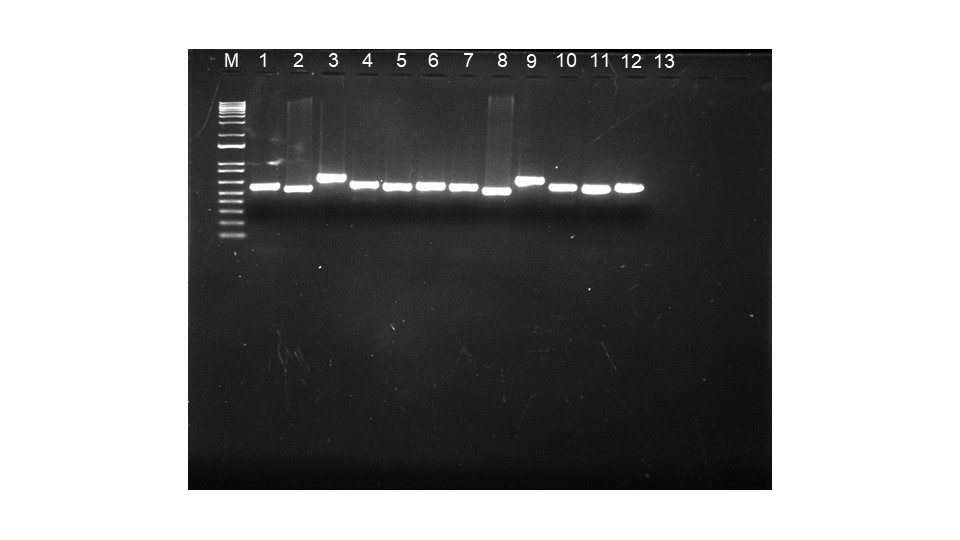

Supplement: Supplementary file 1 [file jof-07-00814-s001.zip › jof-1361809-supplementary/Supplementary Figure S1.jpg]

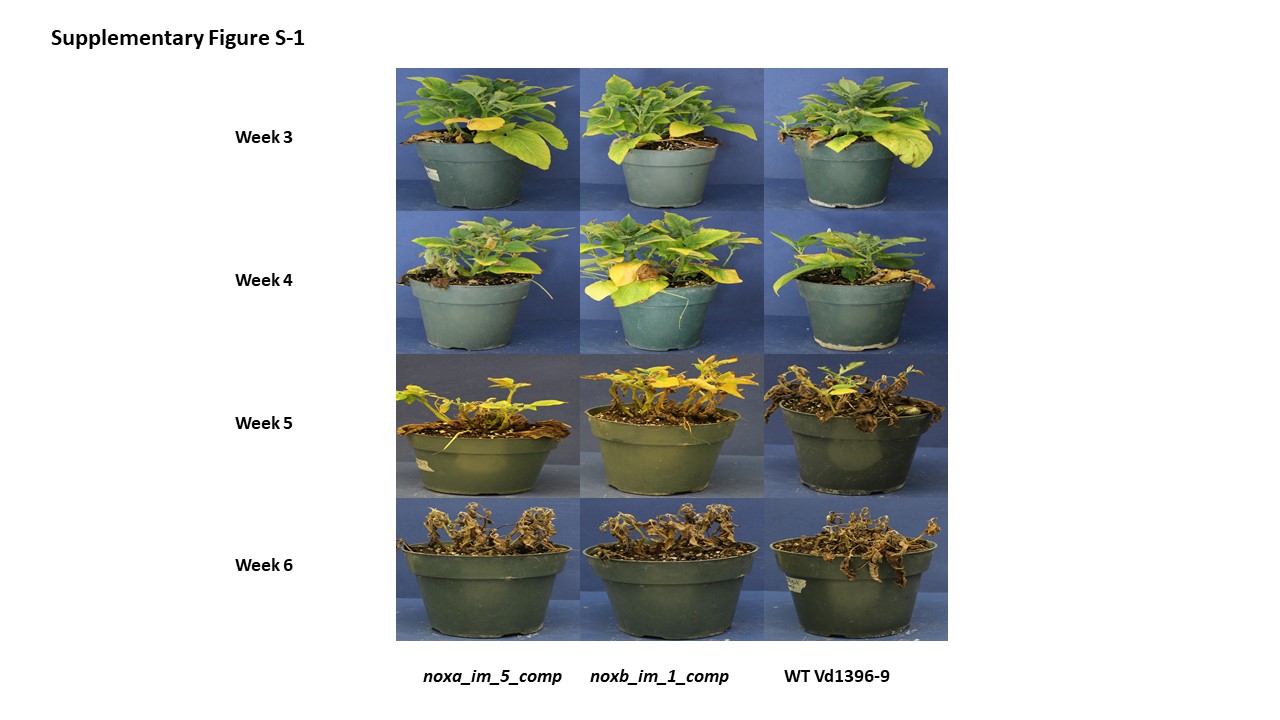

Supplement: Supplementary file 1 [file jof-07-00814-s001.zip › jof-1361809-supplementary/Supplementary Figure S2.jpg]

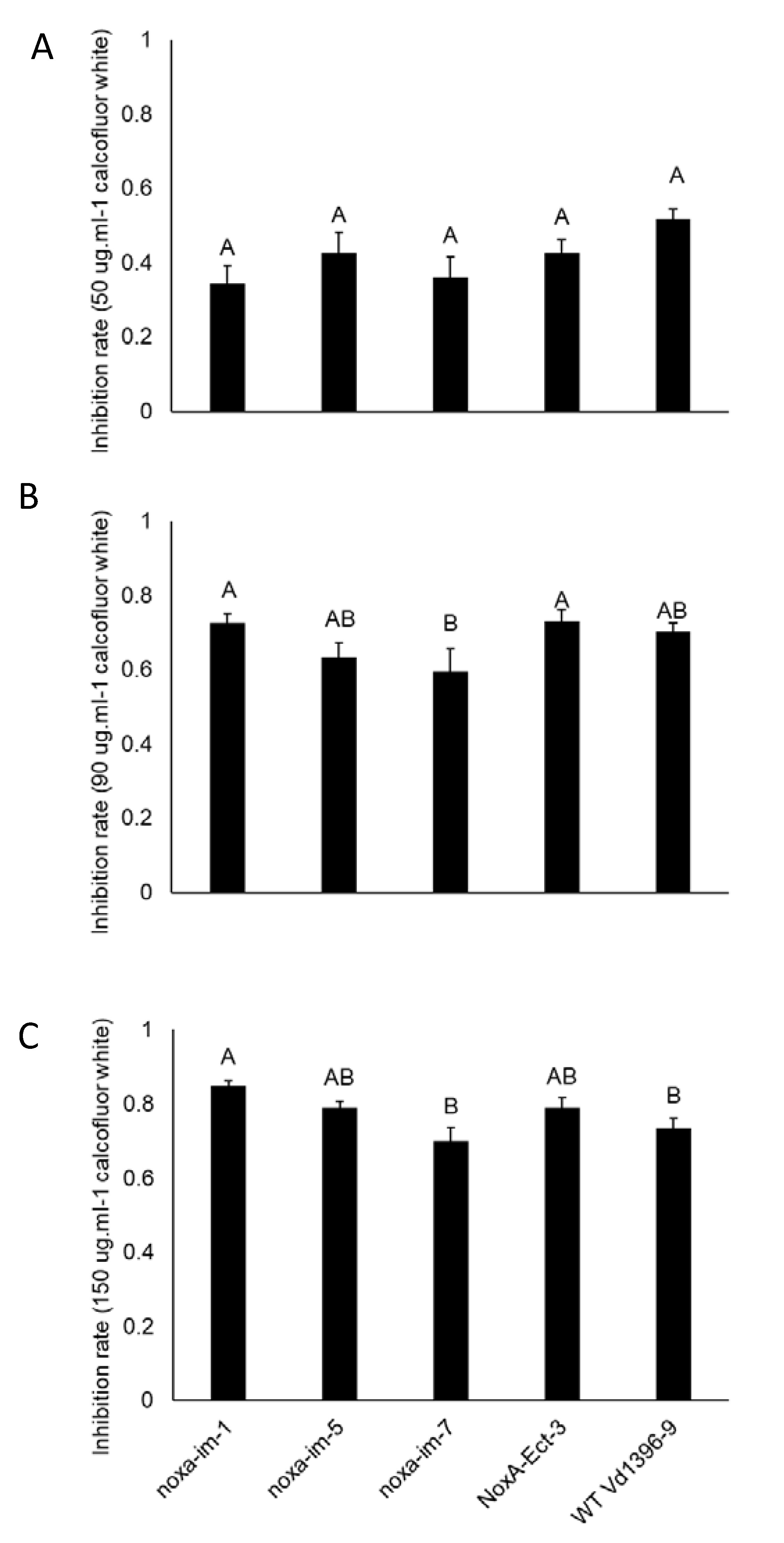

Supplement: Supplementary file 1 [file jof-07-00814-s001.zip › jof-1361809-supplementary/Supplementary Figure S3.jpg]

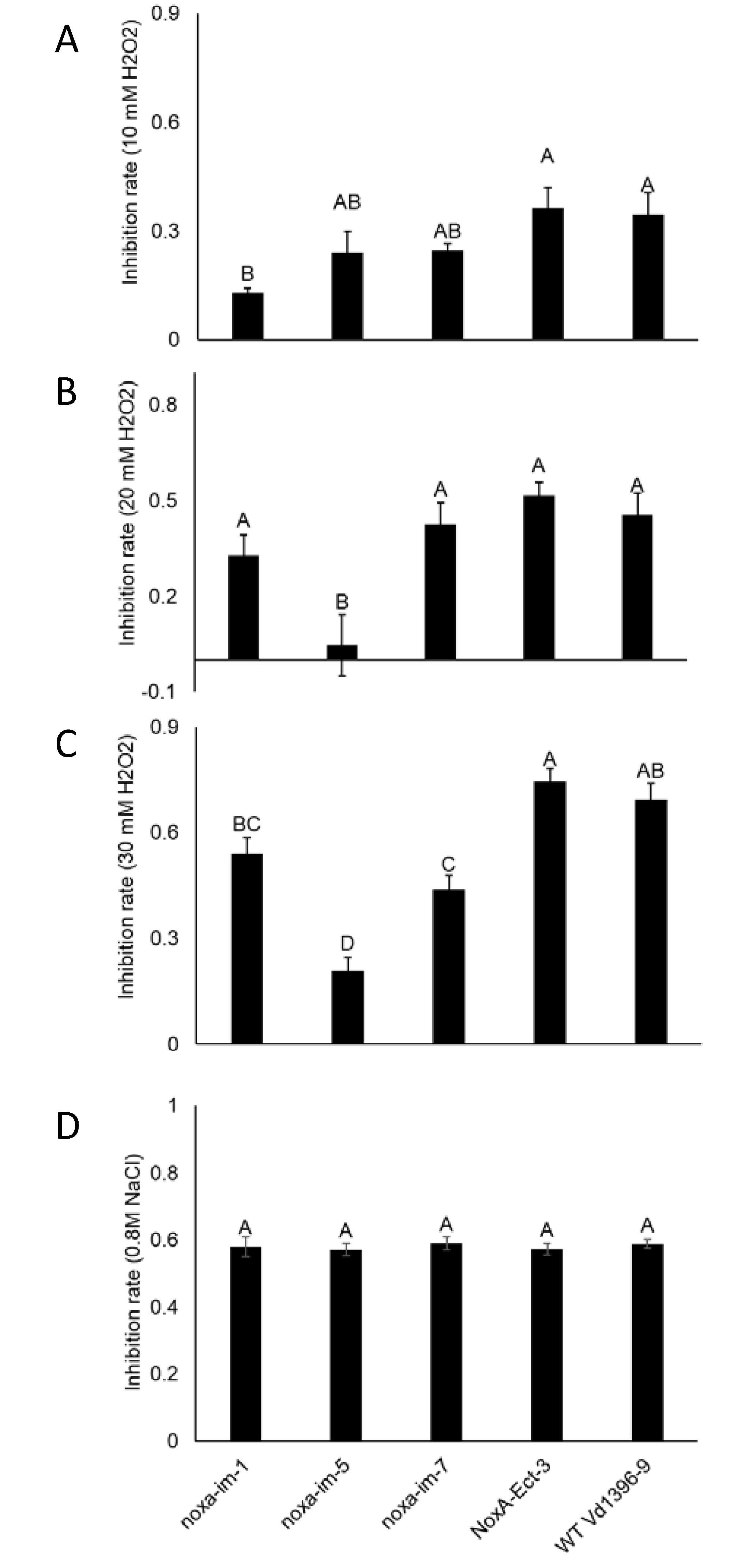

Supplement: Supplementary file 1 [file jof-07-00814-s001.zip › jof-1361809-supplementary/Supplementary Figure S4.jpg]

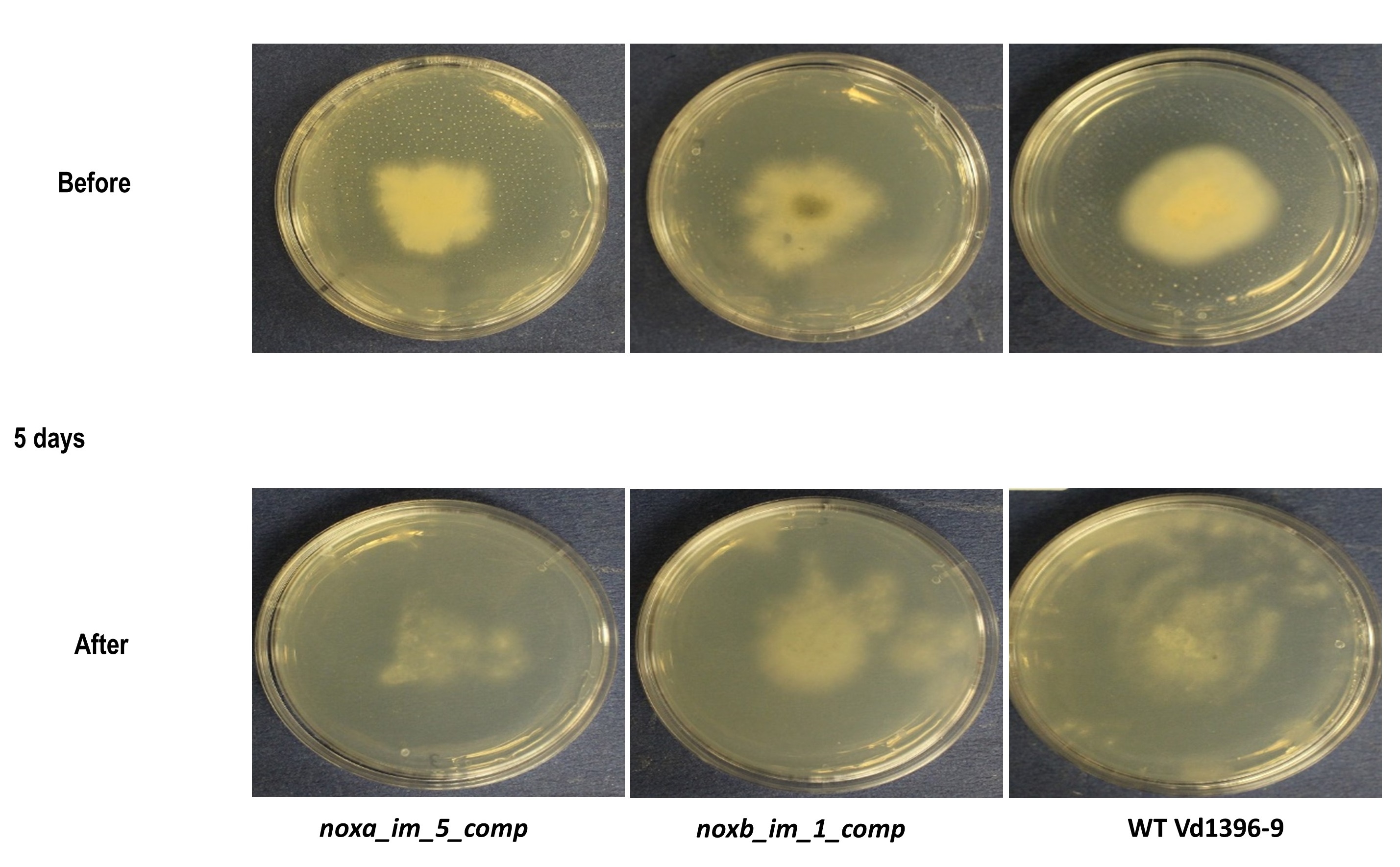

Supplement: Supplementary file 1 [file jof-07-00814-s001.zip › jof-1361809-supplementary/Supplementary Figure S5.jpg]

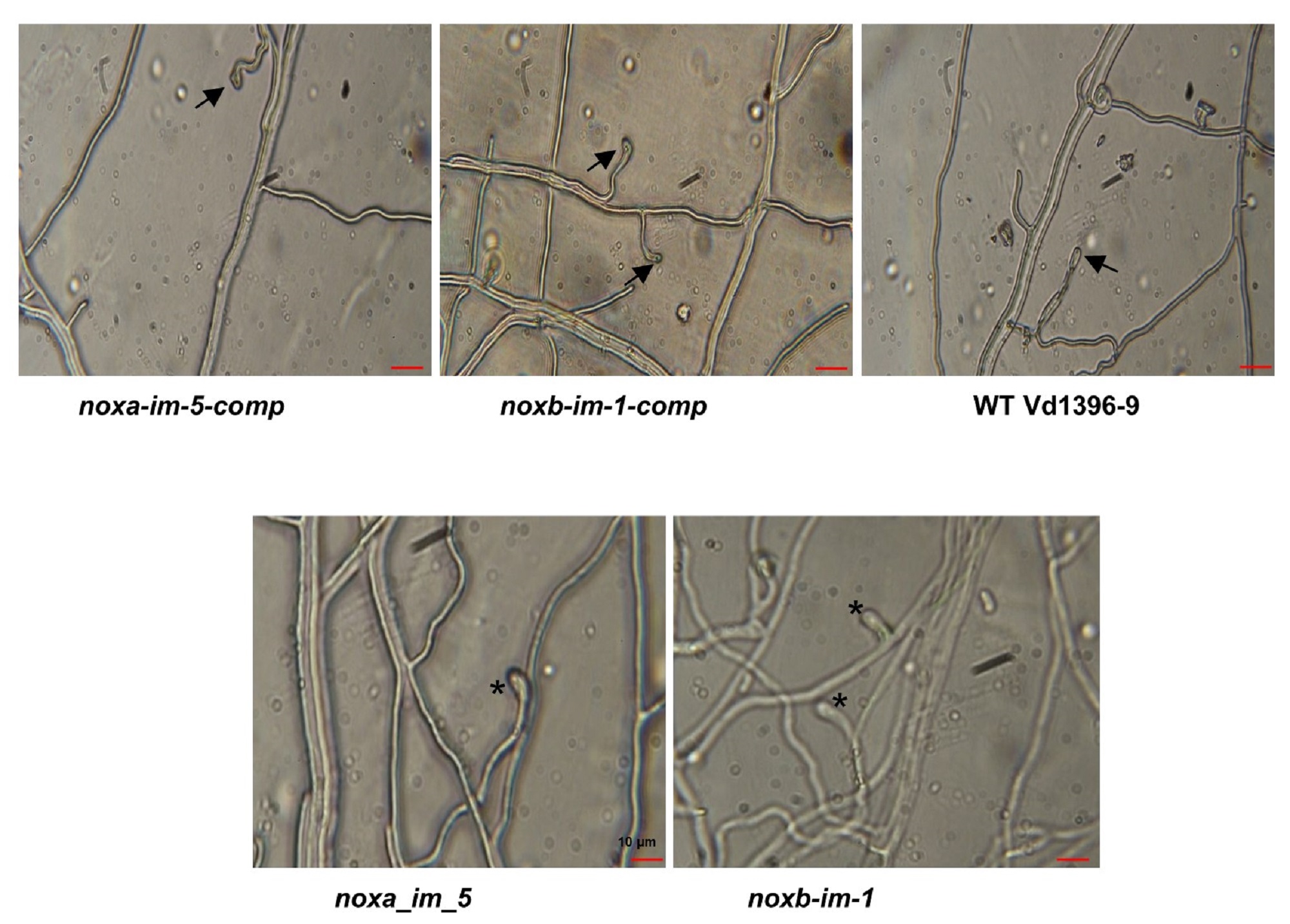

Supplement: Supplementary file 1 [file jof-07-00814-s001.zip › jof-1361809-supplementary/Supplementary Figure S6.jpg]
